# Passive Exposure to Pollutants from a New Generation of Cigarettes in Real Life Scenarios

**DOI:** 10.3390/ijerph17103455

**Published:** 2020-05-15

**Authors:** Joseph Savdie, Nuno Canha, Nicole Buitrago, Susana Marta Almeida

**Affiliations:** 1 Center for Nuclear Sciences and Technologies (C2TN), Instituto Superior Técnico, University of Lisbon, Estrada Nacional 10, 2695-066 Bobadela-LRS, Portugal; jsavdie@gmail.com (J.S.); nunocanha@ctn.tecnico.ulisboa.pt (N.C.); nbuitrago21@gmail.com (N.B.); 2 Centre for Environmental and Marine Studies (CESAM), University of Aveiro, Campus Universitário de Santiago, 3810-193 Aveiro, Portugal

**Keywords:** indoor air quality, e-cigarettes, heat-not-burn tobacco, traditional smoking products, tobacco smoke, passenger cars

## Abstract

The use of electronic cigarettes (e-cigarettes) and heat-not-burn tobacco (HNBT), as popular nicotine delivery systems (NDS), has increased among adult demographics. This study aims to assess the effects on indoor air quality of traditional tobacco cigarettes (TCs) and new smoking alternatives, to determine the differences between their potential impacts on human health. Measurements of particulate matter (PM_1_, PM_2.5_ and PM_10_), black carbon, carbon monoxide (CO) and carbon dioxide (CO_2_) were performed in two real life scenarios, in the home and in the car. The results indicated that the particle emissions from the different NDS devices were significantly different. In the home and car, the use of TCs resulted in higher PM_10_ and ultrafine particle concentrations than when e-cigarettes were smoked, while the lowest concentrations were associated with HNBT. As black carbon and CO are released by combustion processes, the concentrations of these two pollutants were significantly lower for e-cigarettes and HNBT because no combustion occurs when they are smoked. CO_2_ showed no increase directly associated with the NDS but a trend linked to a higher respiration rate connected with smoking. The results showed that although the levels of pollutants emitted by e-cigarettes and HNBT are substantially lower compared to those from TCs, the new smoking devices are still a source of indoor air pollutants.

## 1. Introduction

There is a scientific and medical consensus that cigarette smoking is causally related to lung cancer, heart disease, emphysema and other serious diseases in smokers [1]. Every year, about 8 million people worldwide die from tobacco use [2], and its consumption has been consistently declared as the leading cause of morbidity and mortality in the world [3]. Tobacco smoke is a complex mixture of numerous toxic and carcinogenic substances, containing more than 8000 chemicals produced by distillation, pyrolysis and combustion reactions when tobacco is burnt during both the smoldering and puffing of a cigarette [4].

Convincing scientific evidence has been available for a long time from experimental and epidemiological studies demonstrating that exposure to environmental tobacco smoke (ETS), called secondhand smoke (SHS) or passive smoke, also causes respiratory and heart diseases including lung cancer in adult nonsmokers [5]. In 2017, 1.22 million deaths were caused by SHS [2] (approximately 15% of the deaths linked to tobacco). In children, SHS interferes with lung development, promotes allergic sensitization and asthma, and increases the risk of sudden infant death syndrome [6,7]. The International Agency for Research on Cancer has classified ETS as carcinogenic [5].

Following smoking bans introduced in many countries prohibiting tobacco smoking in public spaces to minimize exposure, the tobacco industry initiated major investments in promoting new (sometimes unregulated) products for consumers. These products were advertised as more appealing than traditional cigarettes (TCs) in terms of social tolerance and health risks. Beliefs that these new products are useful as cessation tools are associated with elevated odds of use in locations where TCs are prohibited [8].

Examples of new smoking products are electronic cigarettes (e-cigarettes), which are battery-powered devices that produce an aerosol from a water-based solution, and heat not-burn tobacco (HNBT), which has been described as a hybrid between TCs and e-cigarettes.

Investigations (some of them developed by the tobacco industry) concluded that although these products are still not entirely safe, they can be considered harmless compared to TCs and, if regulated and controlled, a method to quit addiction to TCs [9,10,11].

Despite these claims, some research results suggest that inhaling propylene glycol-containing e-cigarette aerosols may have adverse health effects, especially in the respiratory and cardiovascular systems [12,13]. Vaping indoors can also release vegetable glycerin, nicotine, aldehydes and heavy metals at levels that may pose a health risk to others [14,15]. In the United States, during 2019, more than 2000 people developed serious lung damage in a poisoning outbreak associated with the use of vaping devices, and 39 people have died from the condition. The United States Centers for Disease Control and Prevention has recently identified vitamin E acetate, an ingredient added to illicit vaping liquids, as the main cause. Recent research has also shown that HNBT produces toxic compounds (e.g., formaldehyde), which are inhaled together with the aerosol [16]. It is also unclear if these new products reduce or increase nicotine addiction [17]. It has been suggested that they can change the epidemiological perception of smoking and likely attract adolescents into smoking dependence [18,19,20].

Due to the increasing popularity of e-cigarettes and HNBT as alternatives to TCs, the World Health Organization (WHO) recognized the importance of monitoring and closely following the evolution of new tobacco products, including products with potentially “modified risks”. There is a need for further documentation and research about the emissions, impacts on indoor air quality, potential health risks for passive smokers and benefits of the new devices [21]. This study evaluated the levels of particles, black carbon, carbon monoxide and carbon dioxide during the smoking of e-cigarettes, HNBT and TCs in homes and cars to assess the potential exposure of smokers and non-smokers.

## 2. Materials and Methods

### 2.1. Sampling Sites Description

**Figure 1:**
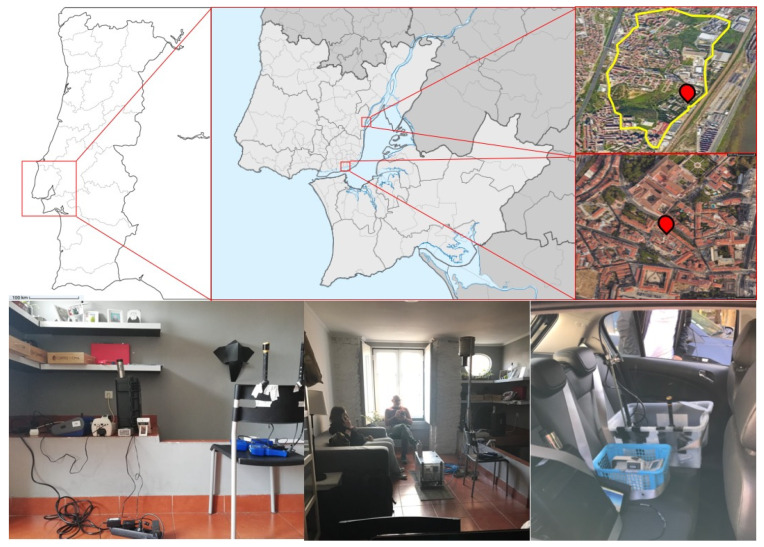
Top: measurement locations within the Lisbon metropolitan area (Portugal)—car route in Loures municipality (**right**, **top**) and location of the studied flat in Lisbon municipality (**right**, **middle**). Bottom: arrangement of the measuring instruments in the home (**left** and **center**) and car (**right**).

**Home measurements** were performed in the sitting room of an occupied flat located in Lisbon, Portugal (Figure 1). The sitting room had a volume of 73 m^3^ and was decorated with typical home furniture. During the experiments, the room was occupied by two people. The air quality monitoring equipment was placed 1.5 m away from the smoker with probes and absorption tubes pointed upwards, at a height of approximately 1 m from the floor. Subjects were told to smoke as usual and not to blow directly onto the equipment.

**Car measurements** were performed inside a medium volume car (Diesel Opel Corsa, from 2007) traveling on a low traffic intensity route of 4.95 km at a mean speed of 34 km/h. The route was located in the municipality of Loures, Portugal, between the neighborhoods of Bobadela and São João de Talha (Figure 1). The real time monitors were placed in the back seat of the car, in open boxes that were fastened with seatbelts to prevent their slipping. The probes or absorption tubes of the various devices were positioned in the area corresponding to the breathing zone of a child. The study was carried out with two occupants in the car: a driver (the smoker) and a non-smoking passenger seated in the front passenger seat.

### 2.2. Smoking Devices

Three different types of NDS were used in this work, all used by volunteer smokers:

**Traditional cigarettes (TC)** are comprised of a blend of dried and cured tobacco leaves which are rolled into a thin rolling paper for smoking. TCs burn at temperatures of around 800 °C, generating smoke that contains harmful chemicals. This work used two types of cigarette of a commonly smoked brand in Portugal, Chesterfield blue (TC1) and Chesterfield menthol (blue caps) (TC2).

**E-cigarettes** are battery-powered devices that produce an aerosol, from a water-based solution, containing a mixture of nicotine, glycerin, propylene glycol and flavoring chemicals, differing depending on the commercial brand. This work used two different types of e-cigarette: the one most common in the USA (JUUL: Slate JUUL, 4.5V, 8W, 5% nicotine pods) and that in Europe (Vape: IStick TC40W, nicotine free liquid)**.**

**Heat-not-burn tobacco (HNBT)** is comprised of a small cigarette made of elements that include a tobacco plug, hollow acetate tube, polymer-film filter, cellulose-acetate mouthpiece filter, and outer and mouth-end papers. It is equipped with electronics that heat specially prepared and blended tobacco, just enough to release a flavorful nicotine-containing vapor but without burning the tobacco. HNBT is heated up to temperatures below 350 °C in an effort to produce lower amounts of air toxicants [22]. This work used the iQOS from Philip Morris International, which is the most popular brand in Europe and America.

### 2.3. Measurement Equipment and Protocol

Continuous measuring portable monitors were used to carry out measurements of indoor concentrations of smoking related pollutants:

The **DustTrack DRX monitor** (8533 model, TSI, Dallas, TX, USA) was used to measure the concentration of particles in a size range between 0.1 to 15 μm. It is a multi-channel, battery-operated, data-logging device, which uses a light-scattering laser photometer that allows the simultaneous measurement of size-segregated mass fraction concentrations corresponding to PM_1_, PM_2.5_, respirable, PM_10_, and total PM size fractions. The resolution of the equipment is ±0.1% of the reading or 0.001 mg/m^3^.

The **CPC TSI 3007** was used to measure the number concentration of ultrafine particles (UFP) with a size range between 0.01 and 1.0 μm (PM_0.01–1_). It operates by drawing an aerosol sample continuously through a heated saturator, in which alcohol is vaporized and diffused into the sample stream. Together, the aerosol sample and alcohol vapor pass into a cooled condenser where the alcohol vapor becomes supersaturated. Here, particles grow quickly into larger alcohol droplets and pass through an optical detector where they are counted. The accuracy of the equipment is ±20%, and the resolution is 0.001 µg/m^3^.

The **MicroAethalometer AE51** (AethLabs, San Francisco, CA, USA) was used to measure black carbon. In the AE51, the air sample is collected by a T60 filter medium (Teflon coated glass fiber). During operation, the microprocessor makes optical measurements, measures and stabilizes the airflow, and calculates the mass concentration of black carbon. The measurement is performed at 880 nm, and the concentration is obtained by the rate of change in the absorption of the transmitted light due to the continuous deposition of black carbon in the filter and the determination of the attenuation of the source light. The measurement precision is ±0.1 µg/m^3^, at a 150 ml/min flow rate, and the resolution is 0.001 µg.

The **TSI 7545** (7545 model, TSI, Dallas, TX, USA) was used to simultaneously measure and log CO, using an electro-chemical sensor, and CO_2_, with a non-dispersive infrared sensor. The accuracy of the CO and CO_2_ concentrations is ±3% of the reading, and the resolution is 0.1 ppm for CO and 1 ppm for CO_2_.

In homes, an initial non-smoking scenario was recorded for 2 hours and used as a control. Afterwards, each NDS was continuously measured for 2 hours divided into eight 15-minute intervals. Each interval consisted of NDS being smoked with 10 “puffs” for 5 minutes leaving a 10-minute decay period between smokes.

**Figure 2:**
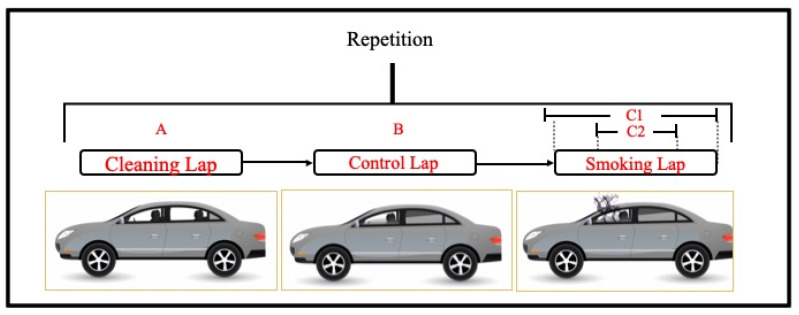
Car measurement methodology.

In cars, the measurement for each NDS was made by completing three repetitions composed of three different individual laps (Figure 2). Lap A consisted of a “cleaning lap” where all windows were open and there was no smoking; Lap B was a “blank/control lap” where all windows were closed except for the driver’s, which was opened halfway, with no smoking; and Lap C consisted of a “smoking lap”, which replicated the conditions of the blank/control lap (all windows closed except for the driver’s) with smoking. During Lap C, measurements were registered separately for the complete lap (measurements C1), which included the pollutants’ decay, and only during the smoking period within the lap, beginning when the cigarette was lit until it was turned off (measurements C2). Each lap lasted between 8 and 10 minutes in which 10 “puffs” were taken per NDS, for an average smoke time of 3 minutes and with a 7-minute decay period. To maintain the external conditions, the study test drives took place outside of the traffic peak period.

### 2.4. Emission Factors

Emission factors for the air pollutants emitted in homes were calculated using Equation (1) [23]:

EF = (C_ave_ * ACH * V)/(n_ave_), (1)

where EF is the emission factor of TCs, e-cigarettes or HNBT in µg/h; C_ave_ is the timed-average pollutant indoor concentrations during the smoking session (µg/m^3^); n_ave_ is the number of TCs, e-cigarettes or HNBT being smoked during the average unit smoking time; ACH is the air change per hour (h^−1^); and V is the room volume (m^3^).

Black carbon concentrations were used to calculate the ACH as it is a conservative and stable pollutant, according to Equation (2) [23]:

ACH = (lnC_ini_ − lnC_end_)/t, (2)

where C_ini_ is the initial concentration of black carbon (ng/m^3^), C_end_ is the final concentration of black carbon (ng/m^3^), t is the total time (h) and ACH is the air change per hour (h^−1^).

### 2.5. Statistical Analysis

The analysis of the variance of the results was performed by non-parametric statistics for a significance level of 0.05. The Mann–Whitney U test was used to test whether two independent groups are likely to derive from the same population, considering the null hypothesis that the two samples have the same median. Therefore, this test assessed whether observations in one sample tend to be larger than observations in the other, such as in the case of air pollutant concentrations associated with the different types of smoking product, the air pollutant levels for the background and smoking periods, and the contribution of the particles’ sizes to the PM_10_ for the different NDS. The statistical calculations were performed using the Statistica software.

## 3. Results and Discussion

### 3.1. Home Scenario

A comprehensive evaluation of the levels of smoking related pollutants in a home while TCs and new smoking products (e-cigarettes and HNBT) were being smoked was performed. The concentrations of the measured indoor air pollutants are summarized in Table 1, and the basic statistics are summarized in Table S1 of the Supplementary Materials. 

#### 3.1.1. Particulate Matter

**Figure 3:**
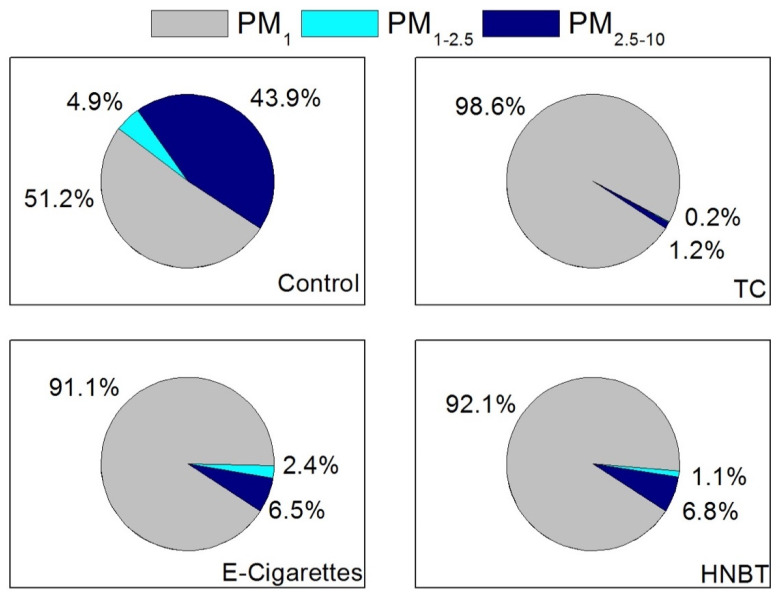
Contribution of each particle size fraction (PM_1_, PM_1–2.5_, PM_2.5–10_) to the PM_10_ in the home discriminated by NDS.

Figure 3 depicts the contribution of each particle size fraction (PM_1_, PM_1–2.5_, PM_2.5–10_) to the PM_10_ for the studied NDS and control. The Mann–Whitney test showed that there was a significant difference between the contributions of the three particle size ranges to the PM_10_ in the non-smoking and NDS trials. PM_1_ was the dominant size fraction for TCs (98.6%), e-cigarettes (91.1%) and HNBT (92.1%) followed by PM_2.5–10_ (TCs: 1.2%, e-cigarettes: 6.5% and HNBT: 6.8%), whereas in the control, the contribution of the coarsest particles to the PM_10_ mass increased to 43.9%.

The use of TCs led to the highest increase in PM_1_ (3470 ± 1570 µg·m^−3^), PM_2.5_ (3480 ± 1570 µg·m^−3^) and PM_10_ (3480 ± 1570 µg·m^−3^) concentrations, followed by the e-cigarettes (PM_1_: 1350 ± 1510 µg·m^−3^; PM_2.5_: 1370 ± 1520 µg·m^−3^; PM_10_: 1380 ± 1520 µg·m^−3^) and HNBT (PM_1_: 80.6 ± 51.3 µg·m^−3^; PM_2.5_: 81.6 ± 51.3 µg·m^−3^; PM_10_: 87.8 ± 51.7 µg·m^−3^). The Mann–Whitney test showed that the concentrations were significantly different between all types of cigarettes and that PM_10_ concentrations measured during the smoking of TCs, e-cigarettes and HNBT were significantly higher than the levels measured in the non-smoking period (165, 64 and 4 times higher, respectively).

Another study on smoke exposure [23] also described higher PM concentrations for TCs than for e-cigarettes and HNBT. However, during the smoking of TCs, Ruprecht et al. [23] obtained PM_1_, PM_2.5_ and PM_10_ concentrations 10, 23 and 2 times lower than those measured in the present study, respectively. Schober et al. [24] also measured lower PM_2.5_ levels associated with the smoking of e-cigarettes (197 µg·m^−3^) than those in the present study.

The differences between the NDS are likely caused by the fact that in TCs, there is a combustion at a temperature <800 °C, which is lower than the temperature needed for complete combustion (around 1300 °C), while e-cigarettes and HNBT are only heated. According to Jiang et al. [25], heating tobacco or e-liquids result in 95% less substances emitted than those produced by the combustion that occurs in TCs. Schober et al. [26] showed that the vaping of the e-cigarettes releases more particles than the use of HNBT. E-cigarette aerosols contain fine and ultrafine liquid particles that are formed from supersaturated propylene glycol vapor, which can penetrate into the respiratory system and cause oxidative stress and inflammatory reactions [27]. Pisinger and Dossing [28] mentioned the irritation of the respiratory tract, evidence of an inflammatory process, a dry cough and an impairment of lung function as short term effects of vaping.

The guidelines defined by the World Health Organization and the limit values according to the Portuguese legislation for indoor air quality (PM_2.5_: 25 µg·m^−3^; PM_10_: 50 µg·m^−3^) were exceeded for TCs (139 and 70 times higher for PM_2.5_ and PM_10_, respectively), e-cigarettes (54 and 27 times higher) and HNBT (3.2 and 1.7 times higher).

**Figure 4:**
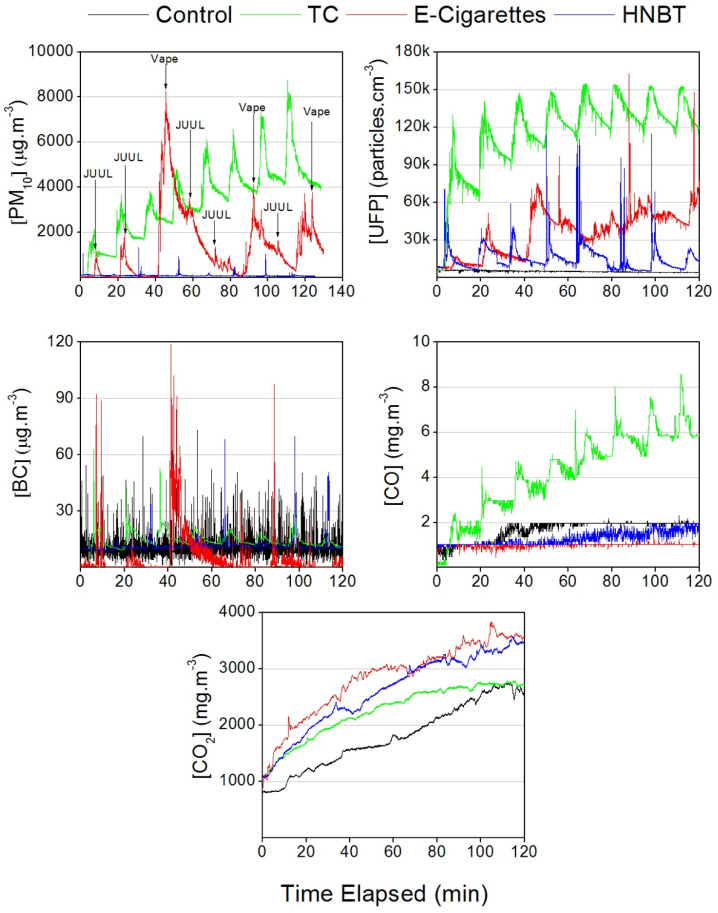
Temporal trends for the PM_10_, UFP, BC, CO and CO_2_ measured during Traditional Cigarettes (TC), e-cigarette and Heat-not-burn tobacco (HNBT) consumption in the home.

Figure 4 shows the temporal trends of PM_10_ levels measured during TC, e-cigarette, and HNBT consumption. The PM_10_ concentrations associated with the TC and e-cigarette trials presented a rapid increase above the background, while for HNBT, the increment was less pronounced but still visible. PM_10_ peaks of more than 8000 µg·m^−3^ were reached for e-cigarettes and TCs. For TCs, PM_10_ levels showed a long decay period, causing an accumulation for each additional cigarette smoked, whereas for both e-cigarettes and HNBT, PM_10_ showed a faster decay and no sign of accumulation. Protano et al. [29] described a similar behavior, since a 1 hour time interval after each smoking each TC was not enough to allow the PM concentration to decrease to the background levels. According to Martuzevicius et al. [30], e-cigarette aerosols have been shown to have a half-life 100 times shorter than TC emissions. The rapid evaporation of liquid droplets from e-liquids is the main reason for the quick decay and the lack of atmospheric accumulation of PM during the use of e-cigarettes.

The PM_1_, PM_2.5_ and PM_10_ emission factors were the highest for TCs, followed by e-cigarettes and HNBT. The emission factors calculated by Ruprecht et al. [23] for TCs were lower for PM_1_ (320 ± 132 µg·min^−1^) than those calculated in this study (844 µg·min^−1^), but higher for PM_2.5_ and PM_10_ (1480 ± 570 and 1540 ± 570 µg·min^−1^, respectively) than the ones calculated here (845 and 846 µg·min^−1^ for PM_2.5_ and PM_10_, respectively). The same study found that both the e-cigarette and HNBT emission factors were non-detectable, significantly differentiating themselves from the elevated values obtained for the present work.

#### 3.1.2. Ultrafine Particles

The Mann–Whitney test showed that the UFP number concentrations were significantly higher during all the smoking sessions than during the background, but the levels for TCs (110,000 ± 36,000 particles·cm^−3^) stood out compared with those for e-cigarettes (37,800 ± 19,000 particles·cm^−3^) and HNBT (35,700 ± 11,500 particles·cm^−3^). The levels for TCs, e-cigarettes and HNBT were 23.4, 8.1 and 7.6 times higher than background, respectively. The UFP concentrations were higher when combustion was occurring i.e., during TC use [25]. This fact explains why both e-cigarettes and HNBT showed lower UFP concentrations compared to TCs.

Atmospheric UFP are mainly composed of organic compounds, trace metal oxides and elemental carbon [31]. Ruprecht et al. [23] found that for selected metals, trace elements and organic compound emission factors varied between TCs, e-cigarettes and HNBT. This means that the type of NDS used highly influences the UFP number concentration. Avino [32] also showed that during a TC test, the increase in the particle number concentration is due to the emission during the smoking activity of particles with a mode of roughly 100 nm, while the e-cigarettes emit particles sized with a mode of about 30 nm.

The UFP number concentrations for TCs and HNBT were similar to those measured by Ruprecht et al. [23] (123,000 ± 37,000 and 27,700 ± 10,300 particles·m^−3^, respectively). For e-cigarettes, Ruprecht et al. [23] measured concentrations 4.4 times lower (8660 ± 560 particles·m^−3^) and Schober et al. [24] obtained concentrations 1.6 times higher (61,700 ± 16,000 particles·m^−3^) than in the present study. The discrepancies found are likely due to high variability in emissions due to the types of equipment and e-liquid being used. Schober et al. [24] used a Red Kiwi (second generation e-cigarette), which is larger and has more wattage than the Elips Series C (second generation e-cigarette) used by Ruprecht et al. [23] and smaller than the third generation e-cigarettes used in this study. Moreover, Zhao et al. [33] also showed that the heating coil temperature, puff duration and puff flow rate in e-cigarettes influence the number concentration of the particles.

The real-time UFP number concentration plot presented in Figure 4 shows an initial cumulative behavior in TCs that reaches a plateau at around 150,000 particles per cm^3^. The UFP temporal pattern for e-cigarettes and HNBT shows a behavior similar to the one obtained by Protano et al. [29], which is characterized by non-accumulation and rapid decay.

The UFP emission factors were the highest for TCs, followed by e-cigarettes and HNBT. The emission factors obtained for TCs, e-cigarettes and HNBT by Ruprecht et al. [23] (130 × 10^10^, 1.1 × 10^10^ and 5.3 × 10^10^ particles per min) were much higher than those obtained in the present study.

#### 3.1.3. Black Carbon

The highest black carbon concentrations were measured while TCs were being smoked (13.2 ± 5.2 µg·m^−3^), followed by e-cigarettes (4.3 ± 10.4 µg·m^−3^) and HNBT (1.2 ± 0.7 µg·m^−3^), which are values approximately 63, 20 and 5.6 times higher than those in the non-smoking trials, considering the values presented in Table 1.

Black carbon particles are produced due to the incomplete combustion of carbon-containing materials [34]. As the tobacco or tobacco-derived products within TCs are burned at temperatures below the 1300 °C threshold needed for complete combustion to occur [25], these NDS have been directly identified as black carbon emission sources [35,36].

On the other hand, probably due to the fact that they evaporate a liquid charge rather than combusting it, studies conducted by van Drooge et al. [37] and Ruprecht et al. [23] have shown no connection between the use of e-cigarettes and black carbon emissions. According to van Drooge et al. [37], the difference between the black carbon concentrations recorded during the non-smoking and e-cigarette smoking scenarios are directly linked to outdoor black carbon concentrations, thus indicating that black carbon is not an emission of the e-cigarette vapor. This is the reason why in the study by Ruprecht et al. [23], the temporal patterns show lower black carbon concentrations during e-cigarette smoking than in the control test, similarly to in the present work.

Figure 4 shows that the black carbon measured during the TC smoking trials presented an initial cumulative behavior, reaching a plateau around 20.0 μg·m^−3^. Both e-cigarettes and HNBT had non-cumulative effects and rapid decays, besides the high spikes observed.

#### 3.1.4. Carbon Monoxide

The Mann–Whitney test shows that the use of TCs led to a significant increase in CO levels in homes to 4.2 ± 1.8 mg·m^−3^, a concentration 2.5 above background levels without smoking. The smoking of HNBT and e-cigarettes had no effect on the CO concentration, as already demonstrated by previous studies [9,24,37], because CO is a byproduct of the incomplete combustion of carbonaceous matter that occurs in TCs [38]. The real time CO concentration plotted in Figure 4 shows that both e-cigarettes and HNBT had a steady, non-cumulative behavior, unlike the TCs, which had a cumulative and incremental behavior without reaching a plateau.

None of the NDS surpassed the guidelines defined by the World Health Organization nor the limit values according to Portuguese legislation (10 mg·m^−3^ for 8 h; 30 mg·m^−3^ for 1 h).

#### 3.1.5. Carbon Dioxide

The CO_2_ concentrations were 2890 ± 660 mg·m^−3^ for e-cigarettes, 2640 ± 680 mg·m^−3^ for HNBT and 2220 ± 520 mg·m^−3^ for TCs; approximately 1.6, 1.5 and 1.2 times higher than control levels, respectively. All the NDS as well as the control scenario (also with two occupants) exceeded the recommended World Health Organization CO_2_ maximum concentration (1800 mg·m^−3^).

The real time CO_2_ measurements (Figure 4) show similarities in the incremental behavior of all the NDS and the control. The concentrations steadily increased, reaching almost double their initial values after one hour and roughly thrice after two hours, indicating that exhalations during NDS use did not increase CO_2_ concentrations in peak increments as with other pollutants. A study conducted by Sadjadi and Minai [39] states that this increase in CO_2_ concentrations is related to an increase in the respiration rate of smokers as a response to inflammation in order to compensate for the decrease in oxygen inhalation during smoking rather than to the emissions originating from NDS use.

### 3.2. Car Scenario

Smoking in the interior of cars is of particular concern for the smoker and other non-smoking passengers, principally for the most susceptible such as children and pregnant woman, because the concentrations of potentially harmful substances are expected to be high due to the reduced volume of the cabin. The mean concentrations measured during the test drives are summarized in Table 2, and for the basic statistics, Table S2 from the Supplementary Materials can be consulted.

#### 3.2.1. Particulate Matter

**Figure 5:**
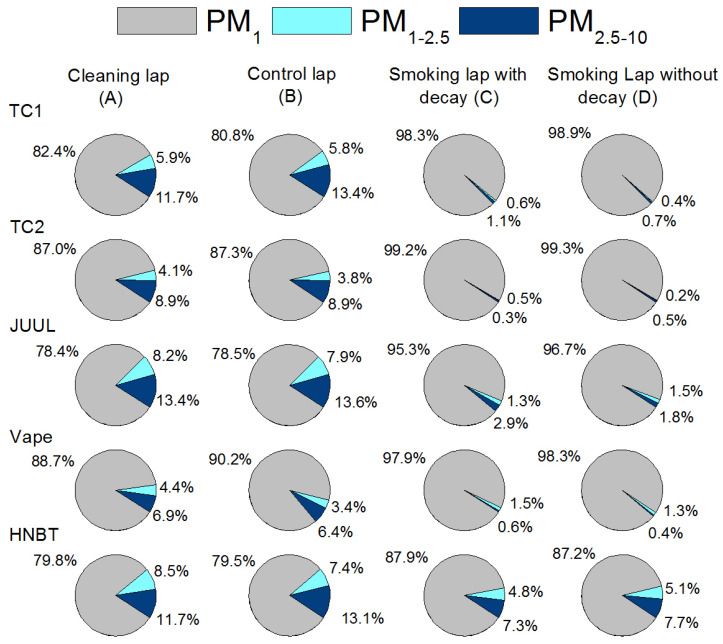
Contribution of each particle size fraction (PM_1_, PM_1–2.5_, PM_2.5–10_) to the PM_10_ in the car discriminated by NDS.

Figure 5 depicts the contribution of each particle size fraction (PM_1_, PM_1–2.5_, PM_2.5–10_) to the PM_10_ for the studied NDS during the different laps. Although no difference was observed between the cleaning and control laps, the Mann–Whitney test indicated a significant difference between the non-smoking and the NDS trials. For the NDS, PM_1_ was the dominant size fraction for TC1 (98.3%), TC2 (99.2%), JUUL (95.3%), vape (97.9%) and HNBT (87.9%), with negligible contributions from the other two fractions. In the control, the two coarser fractions (PM_1__–10_) have a significantly higher contributions during the smoking periods, representing between 9.8% and 21.5% of the PM_10_ mass.

The highest PM_10_ concentrations were measured while the vape was smoked (1170 ± 1160), followed by TC1 (973 ± 597 µg·m^−3^), TC2 (912 ± 881 µg·m^−3^), JUUL (134 ± 190 µg·m^−3^) and HNBT (26.7 ± 22.7 μg·m^−3^). The Mann–Whitney test showed that the PM_10_ concentrations were significantly different for all the types of cigarette except for TC1 and TC2, between which significant differences were not observable.

**Figure 6:**
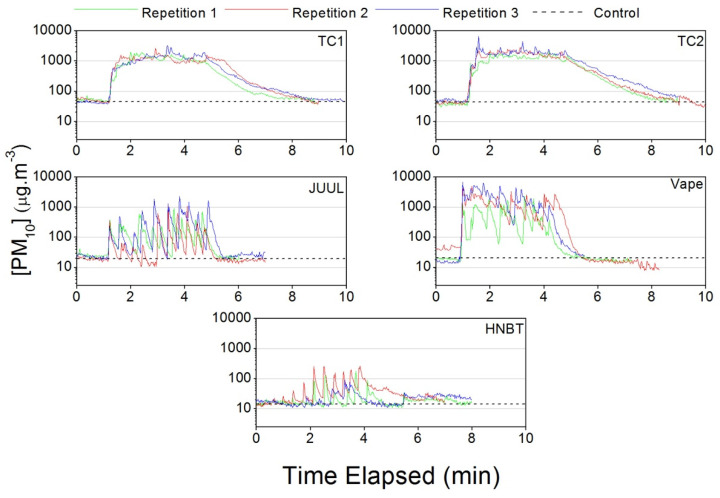
PM_10_ concentrations measured in the car during traditional cigarette (TC1 and TC2), e-cigarette (JUUL and Vape) and heat-not-burn tobacco (HNBT) consumption.

Figure 6 shows the temporal evolution of the PM_10_ concentrations. There is an incremental and cumulative behavior for TC1 and TC2, reaching a plateau at around 1000 µg·m^−3^ before the concentrations start to slowly decrease back to control levels. The JUUL, vape and HNBT time patterns show significant concentration spikes during use but then rapid decreases in concentration.

Geiss et al. [40] measured PM in the vehicle cabins and obtained an average PM_2.5_ concentration in the cars of 26.9 µg·m^−3^, similar to those found in the control level measurements in the present study. Schober et al. [26] studied NDS emissions in seven different vehicles and observed higher mean PM_2.5_ concentrations for TCs (64–1990 µg·m^−3^) when compared to vape (8–490 µg·m^−3^), HNBT (6–34 µg·m^−3^) and control (4–11 µg·m^−3^). In the present study, the e-cigarette vape showed the highest mean levels of PM_2.5_ and PM_10_, even when comparing with TCs.

#### 3.2.2. Ultrafine Particles

The highest UFP concentrations were measured while TC2 were being smoked (142,000 ± 42,000 particles·cm^−3^), followed by TC1 (141,000 ± 56,000 particles·cm^−3^), vape (56,300 ± 39,700 particles·cm^−3^), JUUL (47,800 ± 12,700 particles·cm^−3^) and HNBT (22,100 ± 16,800 particles·cm^−3^). These values are 3.3, 4.4, 3.2, 1.7 and 2.8 times higher than those in the control scenario, respectively.

**Figure 7:**
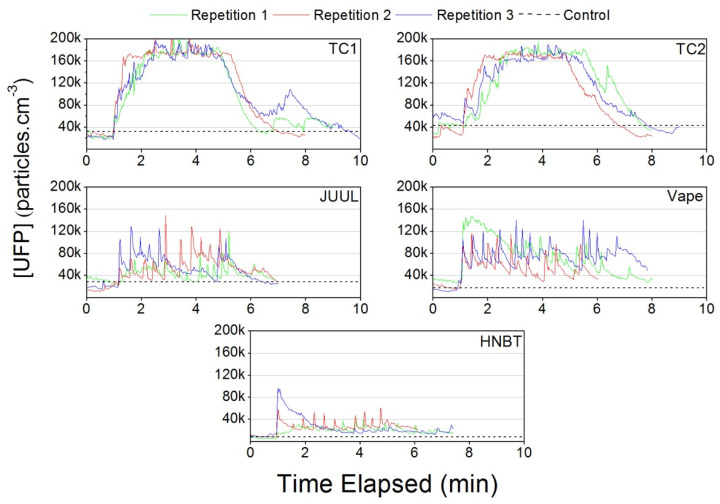
Ultrafine particle concentrations measured in the car during traditional cigarette (TC1 and TC2), e-cigarette (JUUL and Vape) and heat-not-burn tobacco (HNBT) consumption.

TC1 and TC2 showed a longer decay period than the other NDS. Clear spikes were observed for JUUL, HNBT and vapes when “puffs” were taken, but the patterns did not show accumulation and had rapid decays (Figure 7).

The UFP concentrations measured during TC1 and TC2 consumption were significantly higher than for the other NDS, likely due to the combustion that occurred. As previously stated, TCs burn at temperatures of 800 °C, which leads to incomplete combustion, while vape, JUUL and HNBT are only heated. TC1 and TC2 also contain heavy metals and hydrocarbons [41], both of which can be found in the chemical composition of atmospheric UFP [31].

The study developed by Schober et al. [26] showed that TCs also presented the highest UFP levels (ranging from 24,300 to 236,000 particles·cm^−3^), but with HNBT (mean value of 37,900 ± 38,100 particles·cm^−3^, ranging from 16,700 to 124,000 particles·cm^−3^) having higher UFP levels than e-cigarettes (mean value of 31,000 ± 24,100 particles·cm^−3^, ranging from 10,200 to 74,000 particles·cm^−3^) in 71% of the cases.

#### 3.2.3. Black Carbon

The black carbon concentrations were the highest for TC2 (6.1 ± 4.0 µg·m^−3^), followed by TC1 (2.1 ± 0.9 µg·m^−3^), JUUL (1.2 ± 0.6 µg·m^−3^), vape (0.7 ± 1.0 µg·m^−3^) and HNBT (0.5 ± 0.3 µg·m^−3^), representing levels 4.2, 2.5, 2.0, 0.4 and 0.7 times higher than those in the control scenario, respectively. The incomplete combustion that occurs in TCs explains the comparably higher concentrations obtained for this type of NDS.

**Figure 8:**
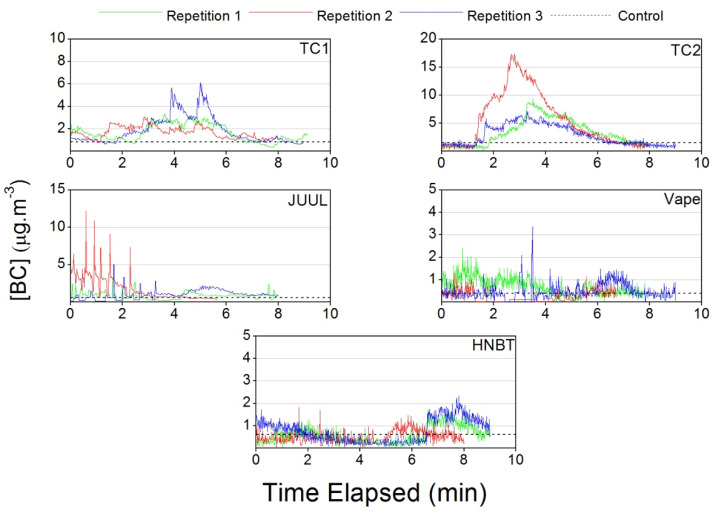
Black carbon concentrations measured in the car during traditional cigarette (TC1 and TC2), e-cigarette (JUUL and Vape) and heat-not-burn tobacco (HNBT) consumption.

The real time black carbon concentrations presented in Figure 8 show an incremental behavior during the use of TC1 and TC2 and a steady decrease after smoking. The JUUL presented a non-cumulative effect, a rapid decay and spikes in concentrations during its use. Both the vape and HNBT patterns showed a non-cumulative effect and rapid decay like the pattern for JUUL, but no spikes in concentrations were observed.

The concentrations in the present study were lower than the black carbon concentrations measured, in vehicles from non-smokers, by Lee et al. [42] (1.9 µg·m^−3^), Cunha-Lopes et al. [43] (5.1 ± 7.3 µg·m^−3^) and Correia et al. [44] (5.5 ± 5.9 µg·m^−3^), except for TC1 and TC2. Onat et al. [45] measured a set of indoor pollutants in different commuting vehicles in Istanbul and registered, for cars, an average black carbon concentration of 2.3 ± 1.3 µg·m^−3^ with closed windows, similar to the results obtained in this study for TC1. Fruin et al. [46] showed that driving behind vehicles in traffic with open windows has a significant effect on the black carbon exposure. This work measured very high levels of black carbon in cars driving behind transit buses reaching up to 92 µg·m^−3^. This would mean that black carbon concentrations in vehicles can be much more related to the outdoor environment rather than to indoor sources, even with a significant emitting source such as an NDS.

#### 3.2.4. Carbon Monoxide

**Figure 9:**
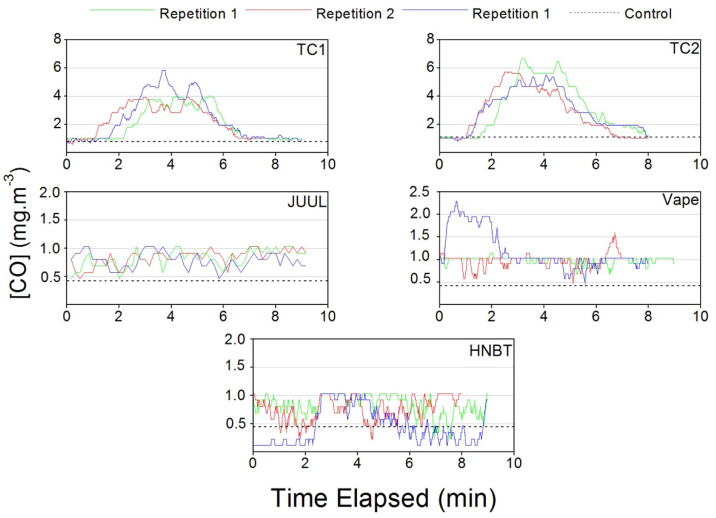
Carbon monoxide concentrations measured in the car during traditional cigarette (TC1 and TC2), e-cigarette (JUUL and Vape) and heat-not-burn tobacco (HNBT) consumption.

Statistical tests showed that the CO concentrations for TC1 (3.0 ± 1.5 mg·m^−3^) and TC2 (4.1 ± 1.6 mg·m^−3^) were significantly higher than for vape (1.1 ± 0.3 mg·m^−3^), JUUL (0.8 ± 0.1 mg·m^−3^) and HNBT (0.7 ± 0.3 mg·m^−3^). Figure 9 shows an incremental and cumulative behavior for TC1 and TC2. E-Cigarettes, JUUL and HNBT show a steady behavior regarding concentrations, with no increases or accumulation occurring during their use. The observed differences are likely linked to the incomplete combustion processes in TC1 and TC2.

Northcross et al. [47] measured CO concentrations in cars during the smoking of TCs and obtained an average concentration of 2.8 ± 1.0 mg·m^−3^ when all windows were half open, while a study conducted by Dirks et al. [48] measured CO concentrations in vehicles ranging from 0.7 to 3.2 mg·m^−^^3^, depending on the window conditions and the ventilation setting in the car.

#### 3.2.5. Carbon Dioxide

The CO_2_ concentrations were the highest during TC2 consumption (1190 ± 50 mg·m^−3^), followed by TC1 (1130 ± 90 mg·m^−3^), vape (1090 ± 60 mg·m^−3^), HNBT (1020 ± 60 mg·m^−3^) and JUUL (982 ± 43 mg·m^−3^).

**Figure 10:**
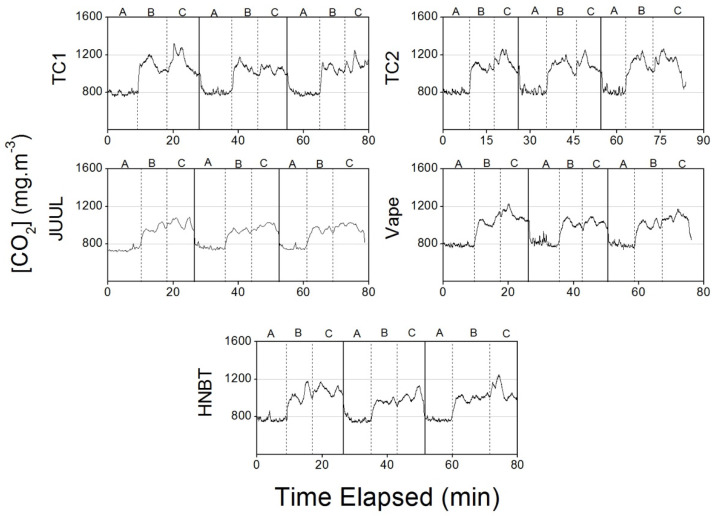
Carbon dioxide concentrations measured in the car during traditional cigarette (TC1 and TC2), e-cigarette (JUUL and Vape) and heat-not-burn tobacco (HNBT) consumption.

Smoking is linked with an increase in respiration rate, which increases CO_2_ concentrations in indoor environments (Figure 10).

Goh et al. [49] measured CO_2_ concentrations ranging between 810 and 1080 mg·m^−3^ in cars, similar to the results for the cleaning laps in the present study. The same study obtained CO_2_ concentrations for two occupants (with all the windows closed) of 2160 mg·m^−3^ nine minutes after the beginning of the experiment. Even without smoking, these values are almost twice the levels measured in the present study for TC2 (1190 mg·m^−3^).

## 4. Conclusions

Although traditional tobacco smoking has been in decline since the 1980s, newer generations of NDS have been steadily increasing in popularity ever since they were introduced into the market in 2013. This accelerated growth, together with their recent appearance, has led to an impendent need for studies to be developed measuring the effects of such.

The present study allowed the evaluation of the concentrations of smoke pollutants, more specifically, the particulate matter and gases originating from different types of NDS in real life scenarios where smoking is still common among electronic nicotine delivery systems users, which consider these a safer option than TCs.

The results showed that although the levels of pollutants emitted by e-cigarettes and HNBT are substantially lower compared to those from TCs, the new smoking devices are still a source of indoor air pollutants. All smoking options are avoidable sources of indoor pollutants, and to protect the health of smokers and non-smokers, they should not be used in homes and cars.

The presented results pertain to a single brand of HNBT and specific brands of e-cigarettes and may not represent the possible variability among different brands or manufacturers. Additionally, the configurations of the equipment as well as the e-liquid charges used for each e-cigarette may not represent other brands or configurations of these devices.

## Figures and Tables

**Table 1:** Air pollutant average concentrations and emission factors for traditional cigarettes (TC), e-cigarettes and heat-not-burn tobacco (HNBT) in the home. NDS, nicotine delivery systems; UFP, ultrafine particles; BC, black carbon.

	**NDS**	**PM_1_ (µg·m^−3^)**	**PM_2.5_ (µg·m^−3^)**	**PM_10_ (µg·m^−3^)**	**UFP (particles·cm^−3^)**	**BC (µg·m^−3^)**	**CO (mg·m^−3^)**	**CO_2_ (mg·m^−3^)**
**Concentrations**	Control	21.0	22.6	25.4	4690	0.21	1.66	1810
	TC	3470	3480	3480	110,000	13.2	4.16	2220
	e-cigarette	1350	1370	1380	37,800	4.30	1.00	2890
	HNBT	80.6	81.6	87.8	35,700	1.18	1.29	2640
**Emission Factors**	**NDS**	**PM_1_ (µg·min^−1^)**	**PM_2.5_ (µg·min^−1^)**	**PM_10_ (µg·min^−1^)**	**UFP (particles·min^−1^)**	**BC (µg·min^−1^)**	**CO (mg·min^−1^)**	**CO_2_ (mg·min^−1^)**
	TC	844	845	846	2.46 × 10^9^	3.37	0.92	604
	e-cigarette	419	424	427	9.89 × 10^8^	1.10	0.26	836
	HNBT	21.9	22.2	23.7	1.20 × 10^9^	0.36	0.33	720

**Table 2:** Air pollutant average concentrations measured in the car for traditional cigarettes (TC1 and TC2), e-cigarettes (JUUL and Vape) and heat-not-burn tobacco (HNBT).

NDS	Lap	PM_1_ (µg.m^−3^)	PM_2.5_ (µg.m^−3^)	PM_10_ (µg.m^−3^)	UFP (particles.cm^−3^)	BC (µg.m^−3^)	CO (mg.m^−3^)	CO_2_ (mg.m^−3^)
TC1	Control	46.2	49.5	57.2	31,733	0.83	0.81	1059
	Smoking	963	967	973	141,000	2.11	3.02	1130
TC2	Control	43.4	45.3	49.7	42,700	1.46	1.10	1090
	Smoking	905	907	912	142,000	6.11	4.12	11,900
JUUL	Control	19.2	21.1	24.5	28,500	0.57	0.43	883
	Smoking	129	131	134	47,800	1.15	0.82	982
Vape	Control	21.0	21.8	23.3	17,600	0.59	0.43	956
	Smoking	1150	1170	1170	56,300	0.70	1.09	1090
HNBT	Control	14.5	15.9	18.3	7940	0.61	0.45	925
	Smoking	23.3	24.7	26.7	22,100	0.46	0.74	1020

## Supplementary Materials

The following are available online at https://www.mdpi.com/1660-4601/17/10/3455/s1, Table S1: Concentrations of air pollutants measured in the home for traditional cigarettes (TC1 and TC2), e-cigarettes (JUUL and Vape), and HNBT, Table S2: Concentrations of air pollutants measured in the car for traditional cigarettes (TC1 and TC2), e-cigarettes (JUUL and Vape), and HNBT.
