# Supplementary material for: Passive Exposure to Pollutants from a New Generation of Cigarettes in Real Life Scenarios
Source: Int J Environ Res Public Health. 2020 May 15;17(10):3455. doi: 10.3390/ijerph17103455 (PMC7277352; doi:10.3390/ijerph17103455)
Supplement: Supplementary file 1 [file ijerph-17-03455-s001.pdf]

Table S1. Concentrations of air pollutants measured in the home for traditional cigarettes (TC1 and TC2), e-cigarettes (JUUL and Vape), and HNBT.

**NDS**		**PM_1 (μg·m⁻³)**	**$PM_{2.5}$ (μg·m⁻³)**	**PM_{10} (μg·m⁻³)**	**UFP (particles·cm⁻³)**	**BC (μg·m⁻³)**	**CO (mg·m⁻³)**	**CO_2 (mg·m⁻³)**
Control	Average	21.0	22.6	25.4	4690	0.21	1.66	1810
	Median	20.0	22.0	24.0	4560	0.13	1.95	1768
	St. Dev.	5.3	5.4	6.9	620	0.31	0.42	580
	Minimum	12.0	14.0	14	2790	0.02	0.23	801
	Maximum	96.0	98.0	98	6250	8.60	2.29	2775
TC	Average	3470	3480	3480	110,000	13.20	4.16	2220
	Median	3630	3630	3630	120,000	13.10	4.58	2393
	St. Dev.	1570	1570	1570	36,000	5.20	1.84	520
	Minimum	50.0	6.0	8.0	5280	0.67	0.11	956
	Maximum	8730	8750	8750	155,000	63.00	8.59	2799
e-cigarette	Average	1350	1370	1380	37,800	4.30	1.00	2890
	Median	843	852	866	41,400	0.80	1.03	4210
	St. Dev.	1510	1520	1520	19,000	10.42	0.08	660
	Minimum	19.0	21.0	22	6270	0.01	0.46	2063
	Maximum	8250	8300	8320	163,000	126.00	1.26	3851
HNBT	Average	80.6	81.6	87.8	35,700	1.18	1.29	2640
	Median	73.0	74.0	81	33,300	1.10	1.26	2799
	St. Dev.	51.3	51.3	51.7	11,500	0.66	0.29	680
	Minimum	40.0	48.0	50	10,100	0.17	0.80	1065
	Maximum	1330	1330	1340	136,000	69.80	1.95	3588

Table S2. Concentrations of air pollutants measured in the car for traditional cigarettes (TC1 and TC2), e-cigarettes (JUUL and Vape), and HNBT.

			PM$_1$ (μg·m^{-3})	PM$_{2.5}$ (μg·m^{-3})	PM$_{10}$ (μg·m^{-3})	UFP (particles·cm^{-3})	BC (μg·m^{-3})	CO (mg·m^{-3})	CO$_2$ (mg·m^{-3})
TC1	Control	Average	46.2	49.5	57.2	31,700	0.83	0.81	1059
		Median	46.0	49.0	56.0	29,300	0.80	0.93	1058
		St. Dev.	10.2	10.4	12.7	13,300	0.33	0.46	67
		Minimum	29.0	32.0	34.0	10,100	0.25	2.0	792
		Maximum	210	214	220	80,100	2.3	3.2	1208
	Smoking	Average	963	967	973	141,000	2.1	3.0	1130
		Median	1080	312	1090	169,000	2.1	1.7	1102
		St. Dev.	597	595	597	56,000	0.90	1.5	90
		Minimum	37.0	40.0	42.0	17,900	0.65	5.9	979
		Maximum	3240	3240	3250	207,000	6.1	5.9	1318
TC2	Control	Average	43.4	45.3	49.7	42,700	1.5	1.1	1090
		Median	40.0	42.0	46.0	34,400	1.3	1.0	1096
		St. Dev.	12.6	12.5	12.9	20,000	0.78	0.23	60
		Minimum	23	24	26	14,900	0.19	0.46	814
		Maximum	120	122	126	104,000	5.0	2.0	1251
	Smoking	Average	905	907	912	142,000	6.1	4.1	1190
		Median	842	844	847	164,000	5.7	4.7	1201
		St. Dev.	880	881	881	42,000	4.0	1.6	50
		Minimum	27.0	29.0	30.0	25,600	0.19	1.0	1082
		Maximum	6490	6500	6500	195,000	17.4	6.7	1264
JUUL	Control	Average	19.2	21.1	24.5	28,500	0.57	0.43	883
		Median	18.0	20.0	23.0	19,600	0.40	0.46	923
		St. Dev.	8.4	8.4	9.4	25,800	0.71	0.27	87
		Minimum	8.0	9.0	10.0	7000	0.01	0.12	738
		Maximum	161	163	206	139,000	1.0	1.0	1021
	Smoking	Average	129	131	134	47,800	1.2	0.82	982
		Median	59.5	61.5	64.0	22,600	0.25	0.81	981
		St. Dev.	190	190	190	12,700	0.60	0.14	43
		Minimum	11.0	12.0	14.0	11,400	0.01	0.58	911
		Maximum	1840	1840	1850	71,600	7.7	1.0	1071
Vape	Control	Average	21.0	21.8	23.3	17,600	0.59	0.43	956
		Median	14.0	14.0	16.0	16,300	0.34	0.23	992
		St. Dev.	28.7	28.8	29.2	7900	2.5	0.37	103
		Minimum	9.0	10.0	10.0	8600	0.02	0.12	758

		Maximum	220	221	224	93,200	1.9	1.9	1093
	Smoking	Average	1150	1170	1170	56,300	0.70	1.1	1090
		Median	829	840	846	35,900	0.87	1.0	1084
		St. Dev.	1150	1160	1160	39,700	0.96	0.29	60
		Minimum	14.0	14.0	14.0	14,100	0.13	0.58	959
		Maximum	6730	6790	6790	147,000	9.1	2.0	1229
HNBT	Control	Average	14.5	15.9	18.3	7940	0.61	0.45	925
		Median	14.0	15.0	17.0	21,600	0.53	0.35	956
		St. Dev.	4.1	4.3	5.9	10,000	0.40	0.33	101
		Minimum	7.0	8.0	8.0	7910	0.05	0.12	734
		Maximum	32.0	35.0	58.0	75,400	0.40	1.0	1170
	Smoking	Average	23.3	24.7	26.7	22,100	0.46	0.74	1020
		Median	17.0	18.0	21.0	17,800	0.41	0.81	1016
		St. Dev.	22.7	22.7	22.7	16,800	0.27	0.29	60
		Minimum	8.0	9.0	10.0	5290	0.04	0.12	817
		Maximum	258	259	263	96,700	1.8	1.0	1139
